# Single- and multiple-trait quantitative trait locus analyses for seed oil and protein contents of soybean populations with advanced breeding line background

**DOI:** 10.1007/s11032-024-01489-2

**Published:** 2024-08-07

**Authors:** Tu Huynh, Kyujung Van, M. A. Rouf Mian, Leah K. McHale

**Affiliations:** 1 Department of Horticulture and Crop Science, The Ohio State University, Columbus, OH 43210 USA; 2 Department of Crop and Soil Sciences, North Carolina State University, Raleigh, NC 270607 USA; 3 Soybean and Nitrogen Fixation Unit, USDA-ARS, Raleigh, NC 27607 USA; 4 Soybean Research Center, The Ohio State University, Columbus, OH 43210 USA

**Keywords:** Single-trait QTL, Multiple-trait QTL, Seed oil content, Seed protein content, Estimated processed values, Soybean breeding

## Abstract

Soybean seed oil and protein contents are negatively correlated, posing challenges to enhance both traits simultaneously. Previous studies have identified numerous oil and protein QTLs via single-trait QTL analysis. Multiple-trait QTL methods were shown to be superior but have not been applied to seed oil and protein contents. Our study aimed to evaluate the effectiveness of single- and multiple-trait multiple interval mapping (ST-MIM and MT-MIM, respectively) for these traits using three recombinant inbred line populations from advanced breeding line crosses tested in four environments. Using original and simulated data, we found that MT-MIM did not outperform ST-MIM for our traits with high heritability (H^2^ > 0.84). Empirically, MT-MIM confirmed only five out of the seven QTLs detected by ST-MIM, indicating single-trait analysis was sufficient for these traits. All QTLs exerted opposite effects on oil and protein contents with varying protein-to-oil additive effect ratios (-0.4 to -4.8). We calculated the economic impact of the allelic variations via estimated processed values (EPV) using the National Oilseed Processors Association (NOPA) and High Yield + Quality (HY + Q) methods. Oil-increasing alleles had positive effects on both EPV_NOPA_ and EPV_HY+Q_ when the protein-to-oil ratio was low (-0.4 to -0.7). However, when the ratio was high (-4.1 to -4.8), oil-increasing alleles increased EPV_NOPA_ and decreased EPV_HY+Q_, which penalizes low protein meal. In conclusion, single-trait QTL analysis is adequately effective for high heritability traits like seed oil and protein contents. Additionally, the populations’ elite pedigrees and varying protein-to-oil ratios provide potential lines for further yield assessment and direct integration into breeding programs.

**Supplementary Information:**

The online version contains supplementary material available at 10.1007/s11032-024-01489-2.

## Introduction

Soybean is among the world’s most important crops and a valuable export commodity of the United States (US). Commercial soybean in the US was valued at $61 billion (Bukowski and Ates 2023). Soybean is a major source of vegetable oil and the primary protein source for animal feed. Therefore, seed oil and protein contents have been crucial traits of focus for many soybean breeding and research programs.

Improving seed oil and protein contents is challenging due to their complex relationships with each other and yield. These traits are negatively correlated, with protein content negatively associated with yield and oil content positively associated with yield (Wilcox and Guodong 1997). Over the past 80 years, breeding programs mainly focusing on yield have consistently resulted in commercial soybean lines with higher yield, increased oil content, and reduced protein content compared to previously released lines (Rinker et al. 2014). These intricate relationships between oil, protein, and yield remain challenging to modulate and continue to drive research efforts to better understand and manipulate them to develop improved cultivars.

Despite this obstacle, protein and oil contents express high heritability levels, facilitating genetic studies and offering an advantage to breeding approaches (Brzostowski et al. 2017; Chung et al. 2003). Over 30 years, 600 + genetic regions linked to these traits have been identified through quantitative trait locus (QTL) analysis (compiled in Van and McHale 2017). Such QTL studies helped confirm and pinpoint genes and gene variants controlling seed oil and protein contents. Seed oil and protein QTLs have been mapped to every soybean chromosome (Chr), and major seed protein QTLs have been repeatedly reported to a region on LG E (Chr 15) and on LG I (Chr 20) (Brummer et al. 1997; Chung et al. 2003; Diers et al. 1992; Fasoula et al. 2004; Jun et al. 2008; Lee et al. 1996; Van and McHale 2017; Warrington et al. 2015). Via fine mapping and gene characterization of the protein-associated locus on Chr 20, variation within a major gene with a significant impact on protein content, *Glyma.20G085100*, was found to be the basis of this important protein QTL (Fliege et al. 2022). The sugar transporter gene *GmSWEET39* has also been characterized to regulate oil and protein content and is potentially the major gene behind the oil and protein QTL on LG E (Chr 15) (Zhang et al. 2020).

Previous soybean QTL studies focused on single-trait analysis, mapping one trait at a time. However, for correlated traits like oil and protein content, multiple-trait QTL analysis has proven statistically more powerful and precise in detecting and mapping QTLs compared to a collection of separate single-trait analyses. The multiple-trait analysis incorporates trait correlations into the model and considers the common QTL effect on multiple traits (Costa E Silva et al. 2012; Jiang and Zeng 1995; Malosetti et al. 2008; Zhu and Zhang 2009). This approach has been widely used in studies of complex correlated traits in biomedical research and animal and plant breeding (Malosetti et al. 2008; Neuschl et al. 2007; Thomasen et al. 2008), yet has not been applied to the correlated traits of seed oil and protein content in soybean breeding.

In this study, we conducted both single-trait and multiple-trait QTL analysis for seed oil and protein contents using three recombinant inbred line (RIL) populations derived from crosses between advanced breeding lines grown in four environments. We applied and compared single-trait multiple interval mapping (ST-MIM) and multiple-trait multiple interval mapping (MT-MIM). Based on the previous claim that the multiple-trait analysis can improve power and precision compared to the single-trait approach, we hypothesized that MT-MIM would outperform ST-MIM and identify more QTLs with minor effects. We detected seven ST-MIM QTLs for oil or protein contents, only five of which were confirmed by MT-MIM, contrary to our prediction. We further hypothesized that the high heritability of these traits led to ST-MIM outperforming MT-MIM. Our simulation study supported this hypothesis showing that for high-heritability traits like seed oil and protein contents, MT-MIM did not provide any added values, and ST-MIM was sufficient to detect QTLs with both major and minor effects. We also calculated the allelic effects on the traits and the estimated processed values. We identified potential candidate genes involved in seed oil and protein biosynthesis and storage within the QTL intervals, providing insights for further characterization and implementation for cultivar development.

## Materials and methods

### Plant materials and experimental design

Three F_5_-derived RIL populations were created from the following crosses: (1) H09-730 × N09-2516 (population 009), (2) H09-018 × N09-2495 (population 023), and (3) ‘Summit’ x N09-2516 (population 037). High-yielding cultivar Summit and advanced breeding lines H09-730 and H09-018 are adapted to Ohio, while the high oil advanced breeding lines N09-2516 and N09-2495 are adapted to North Carolina conditions. In total, 355 F_5_-derived recombinant inbred lines (RILs) (140 in population 009, 103 in population 023, and 112 in population 037) that could be phenotyped in both Ohio and North Carolina field environments were used for this study. Three generations of the RIL populations, F_5:7_ to F_5:9_, were planted between mid-May and early June in four environments: 2017, 2018, and 2019 in Columbus, Ohio, and 2019 in Raleigh, North Carolina. In each environment, 35 seeds of each RIL were planted in a single-row plot in a randomized complete block design with two replicates per RIL. Three Ohio-adapted public cultivars, ‘Lorain’, ‘Summit’, and ‘Clermont’ were used as checks.

### Phenotypic data collection

Plants were combine-harvested between late October and early November of each year, and seeds were further cleaned to remove unwanted materials. Non-green and non-chipped seeds were randomly sampled from each plot for oil and protein content measurement via near-infrared radiation (NIR) spectroscopy using the DA 7250 Near Infrared Analyzer Spectrometer (Perten Instruments®, Hägersten, Sweden). Total seed oil and protein contents were recorded as mass in grams per kilogram of seed weight (g/kg) (Table S1).

### Phenotypic data analysis

The average values for oil and protein contents of each line were calculated as least squares (LS) means, and analysis of variance was conducted using the following statistical model:

$${Y}_{ijk}=\mu +{G}_{i}+{E}_{j}+{R(E)}_{jk}+{GE}_{ij}+{\varepsilon }_{ijk}$$

where *μ* is the overall mean, *G*_*i*_ is the effect of the *i*th line, *E*_*j*_ is the effect of the *j*th environment, *R(E)*_*jk*_ is the effect of the *k*th replication in the *j*th environment, *GE*_*ij*_ is the effect of the interaction between the *i*th line and the *j*th environment, and *ε*_*ijk*_ is the experimental error. For LS means calculation, the genetic background was treated as fixed effects and the remaining factors were random effects. For the analysis of variance, all effects were random. Variance components were extracted for each effect in the model.

Broad-sense heritability (H^2^) on an entry-mean basis was derived by dividing the genetic variance by the total variance for each trait via the formula

$${H}^{2}= \frac{{{\sigma }^{2}}_{G}}{{{\sigma }^{2}}_{G}+ \frac{{{\sigma }^{2}}_{GE}}{{n}_{E}} +\frac{{{\sigma }^{2}}_{\varepsilon }}{{n}_{E}\bullet {n}_{R}}}$$

where $\sigma$ ^2^_G_ is the genetic variance, $\sigma$ ^2^_GE_ is the variance of genetic and environment interaction (GEI), $\sigma$ ^2^_E_ is the residual variance, n_E_ is the number of environments, and n_R_ is the number of replications within an environment.

Pearson’s correlation test was performed to calculate the correlation coefficient from the LS means values of the two associated traits. Pearson’s correlation test was also used to determine the partial genetic correlation between the two traits of protein and oil contents via the formula

$${r}_{m,n}= {\sigma }_{{G}_{\left(m,n\right)}}\sqrt{{\sigma }_{{G}_{m}}^{2} {\sigma }_{{G}_{n}}^{2}}$$

where ${\sigma }_{{G}_{\left(m,n\right)}}$ is the genetic covariance between the two traits m and n, and ${\sigma }_{{G}_{m}}^{2}$ and ${\sigma }_{{G}_{n}}^{2}$ are the genetic variances of each trait. The genetic covariance ${\sigma }_{{G}_{\left(m,n\right)}}$ was calculated via the expression

$${\sigma }_{{G}_{\left(m,n\right)}}= \frac{{{\sigma }_{{G}_{m+n}}^{2}- \sigma }_{{G}_{m}}^{2} {- \sigma }_{{G}_{n}}^{2}}{2}$$

with are the genetic variances of traits m plus n, m, and n, respectively (Alves et al. 2018).

### Genotypic data collection

DNA was extracted from F_5_ generation trifoliate leaf tissues using the Qiagen Plant DNeasy Extraction Kit (Qiagen, Redwood City, CA). Subsequently, DNA samples from the three populations underwent library preparation and genotyping by sequencing (GBS) at the Molecular and Cellular Imaging Center at Ohio Agricultural Research and Development Center (Wooster, OH) via NovaSeq 6000 SP paired-end platform with 150 bp read length. Restriction enzyme *ApeKI* was used to digest genomic DNA for library preparation.

### Genotypic data analysis

Sequence reads underwent quality control using FastQC version 0.11.5 and MultiQC version 1.11 (Andrews 2013; Ewels et al. 2016). BBMap version 38.95 was utilized for trimming adapters and low-quality sequences using the parameters trimq = 15, maq = 15, qtrim = r, minlen = 50, and tbo = t (https://sourceforge.net/projects/bbmap/). High-quality reads passing the post-trimming quality control were subjected to alignment using the Burrows-Wheeler Aligner (BWA) tool version 0.7.17-r1198 and indexing using SAMtools version 1.10 (Danecek et al. 2021; Li and Durbin 2009). The genome version Wm82.a2.v1 (file “Gmax_275_v2.0.fa.gz”, www.phytozome.org) was used as the reference for alignment. The aligned data of each population was then used for variant calling via the SAMtools/mpileup version 1.14. Prior to data imputation, SAMtools/bcftools version 1.14 was also used to process and filter the variant calling file of each population. Samples with more than 60% missing data were removed. Markers with minimum read length (minDP) less than 5, with more than one sample containing a third allele (non-biallelic), with heterozygosity of over 20%, with minor allele frequency (MAF) below 5%, and with more than 85% of missing data were also removed from the dataset for each population. Filtered data underwent imputation for missing parental data via R/MPRgenotyping (Xie et al. 2010) and then imputation for all missing data within the population via R/LaByRInth (Low-coverage Biallelic R-package Imputation, source code at www.github.com/Dordt-Statistics-Research/LaByRInth), which is the R equivalent of the Java-based LB-Impute algorithm (Fragoso et al. 2016). For LaByRInth settings, generation was set as 5 for F_5_, genotype error as 0.015, parent heterozygosity as 0.005, and minimum posterior quality control threshold as 0.85. Post imputation, markers that were non-biallelic had a heterozygosity proportion over 10%, with minor allele frequency (MAF) below 10% were removed using TASSEL 5 (Bradbury et al. 2007). Associated SoySNP50K iSelect BeadChip markers were shown where present based on the physical location of the GBS polymorphisms (Song et al. 2013).

### Genetic map construction

Imputed markers with MAF over 30% and heterozygosity below 10% were used for genetic mapping. Markers totaling 4914, 3485, and 4337 were used for populations 009, 023, and 037, respectively. JoinMap4 was used for constructing genetic maps using genetic data originating from a single chromosome at a time to ensure markers from different chromosomes were not incorrectly grouped into one linkage group (LG). The Kosambi regression model and a minimum LOD score of 2 were used to construct linkage groups.

### Single-trait and multiple-trait quantitative trait locus analyses

The same dataset was used for both single-trait and multiple-trait QTL analysis. Single-trait analysis employed ST-MIM (Kao et al. 1999), while multiple-trait analysis used MT-MIM (Joehanes 2009) in QGene version 4.4.0 (Joehanes and Nelson 2008). A 1-cM scan interval was applied, and the LOD threshold was determined as the 95th percentile from 1000 rounds of permutation. The correlated oil and protein traits were coupled during resampling. Additive effects and LOD scores for each trait and multiple or joint traits were extracted. Loci with LOD scores exceeding the LOD threshold were considered significant. The R package "qtl" was utilized to calculate the phenotypic variance explained by each QTL using the *fitqtl* function, and the function *lodint* was used to determine the 1.5-LOD confidence interval (Broman et al. 2003).

### Comparison of MT-MIM and ST-MIM of simulated datasets with various heritability levels

 The simulation was replicated 20 times. For each replication, R/qtl functions *sim.map* and *sim.cross* were used to randomly generate a RIL genetic map for a population of 300 individuals. The genetic map consisted of six chromosomes, each 80 cM long, with markers evenly spaced every 10 cM. Phenotypic data were simulated for each genetic map using Qgene version 4.4.0 (Joehanes and Nelson 2008) at four heritability levels: 0.3, 0.5, 0.7, and 0.9, corresponding to scenarios HET0.3, HET0.5, HET0.7, and HET0.9. Across all simulations, the populations exhibited an average oil content of 22%, an average protein content of 41%, and a correlation coefficient of -0.7 between oil and protein. Five distinct oil- and protein-associated QTLs, denoted as qSIM-1 to -5, with varying effect size combinations were simulated. Each QTL was placed on a different chromosome to prevent mutual influence. Table 1 shows a summary of the simulated QTLs and scenarios.

**Table 1:** Allelic effects of five simulated QTLs at different heritability levels

Trait	Allelic effects (g/kg)				
	qSIM-1	qSIM-2	qSIM-3	qSIM-4	qSIM-5
Oil	-2.5 (minor)	-2.5 (minor)	-15 (major)	-2.5 (minor)	0 (none)
Protein	2.5 (minor)	15 (major)	15 (major)	0 (none)	15 (major)

MT-MIM and ST-MIM were conducted to assess their performance in detecting these QTLs. A QTL was deemed statistically significant when it resided within a 15 cM proximity of the original QTL and its LOD score exceeded the threshold determined through permutations. The probabilities of identifying a simulated QTL as significant were recorded for both MT-MIM and ST-MIM in each heritability scenario.

### Calculation of allelic effects on estimated processed values

Estimated processed values (EPV) per bushel were calculated using two pricing methods: one from the National Oilseed Processors Association (NOPA) and another called the High Yield + Quality (HY + Q) method via a previously developed R module (USB 2019; Swanson 2021). The R module’s default baseline prices were updated to $353.02 per ton for meal protein content and $0.4256 per pound for oil content to reflect the most recent five-year averages (2017/2018 to 2021/2022) reported by the USDA Economic Research Service (Bukowski and Ates 2023). Mean values of oil and protein contents of each allele were used to calculate the allelic effect on EPV.

### Identification of candidate genes

The physical interval marked by the flanking markers of the 1.5 LOD confidence interval was used for candidate gene search using information from the Wm82.a2.v1 reference genome (www.soybase.org). The confidence interval was expanded to the nearest marker, and the physical location of these markers defined the gene search range. Genes within this range were extracted from the Wm82.a2.v1 genome browser tool on SoyBase, and their functions were obtained using SoyBase’s Gene Annotation Lookup tool and the SoyCyc Metabolic Database (www.soybase.org, Grant et al. 2010). From the resulting annotations, genes related to oil and protein production and accumulation processes, such as fatty acid biosynthesis and beta-oxidation, glycolysis, gluconeogenesis, lipid storage and transport, amino acid biosynthesis and transport, etc., were filtered and considered candidate genes.

## Results

### Seed oil and protein contents frequency distribution, correlation, variance components, and heritability

**Fig. 1:**
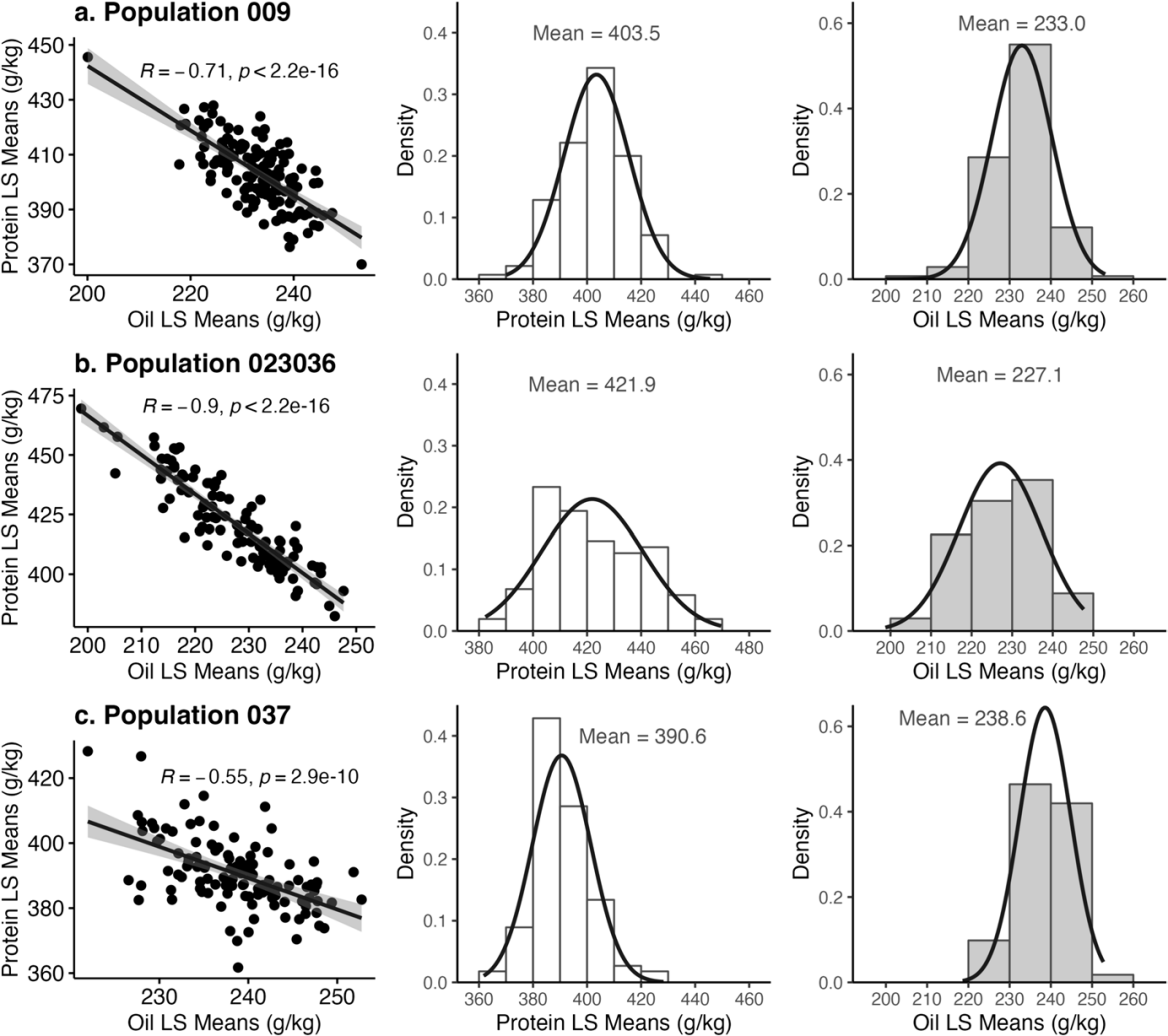
Correlation and distribution of seed oil and protein contents. Total seed protein and oil contents were recorded in grams per kilogram of seed weight (g/kg). Each row represents each of the three populations, including a) 009, b) 023, and c) 037. Pearson’s correlation test was used to retrieve correlation coefficient (R) values and the significance of the correlated relationship. A p-value below 0.05 indicates a significant correlation. LS Means: Least squares means

As predicted, the negative correlation between oil and protein contents was significant and varied across populations. Correlation coefficient values were -0.71, -0.90, and -0.55 for populations 009, 023, and 037, respectively (Fig. 1). The partial genetic correlation between two traits represents the relationship of how one trait varies with the other due to the degree of common genetic control that the two traits share. The partial genetic correlation between protein and oil contents was statistically significant and high in all three populations, with populations 009, 023, and 037 displaying coefficients of -0.75, -0.90, and -0.71, respectively (Table 2).

**Table 2:** Variance component and significance of factors contributing to oil and protein content phenotypes

Population and trait	Variance component ± standard deviation				Broad sense heritability	Partial genetic correlation (Pearson’s correlation)
	Genotype	Environment	GEI	Replicate within Environment		
Pop 009 Protein	1.33 ± 1.15 ****	3.70 ± 1.92 ****	0.44 ± 0.66 ****	0.44 ± 0.66	0.85	-0.75 ****
Pop 009 Oil	0.46 ± 0.68 ****	0.73 ± 0.85 ****	0.16 ± 0.39 ****	0.57 ± 0.75	0.86	
Pop 023 Protein	3.11 ± 1.76 ****	4.10 ± 2.02 ****	0.44 ± 0.66 ****	0.27 ± 0.52	0.93	-0.90 ****
Pop 023 Oil	0.90 ± 0.95 ****	0.60 ± 0.78 ****	0.111 ± 0.33 ****	0.07 ± 0.26	0.94	
Pop 037 Protein	1.25 ± 1.12 ****	4.06 ± 2.01 ****	0.50 ± 0.70 ****	1.25 ± 1.12	0.84	-0.71 ****
Pop 037 Oil	0.42 ± 0.65 ****	0.55 ± 0.74 ****	0.09 ± 0.30 ****	0.26 ± 0.51	0.87	

Asterisks marks (*) indicate significance level based on *p*-values of each component: no asterisk for *p*-value > 0.05 (not significant), * for *p*-value ≤ 0.05, ** for *p*-value ≤ 0.01, *** for *p*-value ≤ 0.001, **** for *p*-value ≤ 0.0001

For population 009, protein and oil contents ranged from 370.0–445.6 g/kg and 200.0–253.2 g/kg, averaging 403.5 g/kg and 233.0 g/kg, respectively. For population 023, protein and oil contents ranged from 382.4–469.5 g/kg and 198.8–247.6 g/kg, averaging 421.9 g/kg and 227.1 g/kg, respectively. For population 037, protein and oil contents ranged from 361.7–428.3 g/kg and 222.0–252.7 g/kg, averaging 390.6 g/kg and 238.6 g/kg, respectively. Distributions of oil and protein contents were relatively normal (Fig. 1).

The effect of genotype-by-environment interaction (GEI) was significant in all three populations for both oil and protein contents. Significance was also observed for genotypes and environment (Table 2). Broad-sense heritability was high for both traits across all populations, ranging from 0.84 to 0.93 for protein and 0.86 to 0.94 for oil (Table 2).

### SNP-based genetic maps

A total of 4914, 3485, and 4337 markers were used to build a genetic map via JoinMap4 using the Kosambi regression model for populations 009, 023, and 037, respectively (Tables S2-S4; Ooijen et al. 2006). Genetic maps representing all 20 chromosomes were constructed, with some chromosomes represented by more than one linkage group for each population. The genetic maps of populations 009, 023, and 037 were comprised of 33, 32, and 30 linkage groups, respectively (Tables S2-S4).

### Single-trait and multiple-trait QTL analyses

**Fig. 2:**
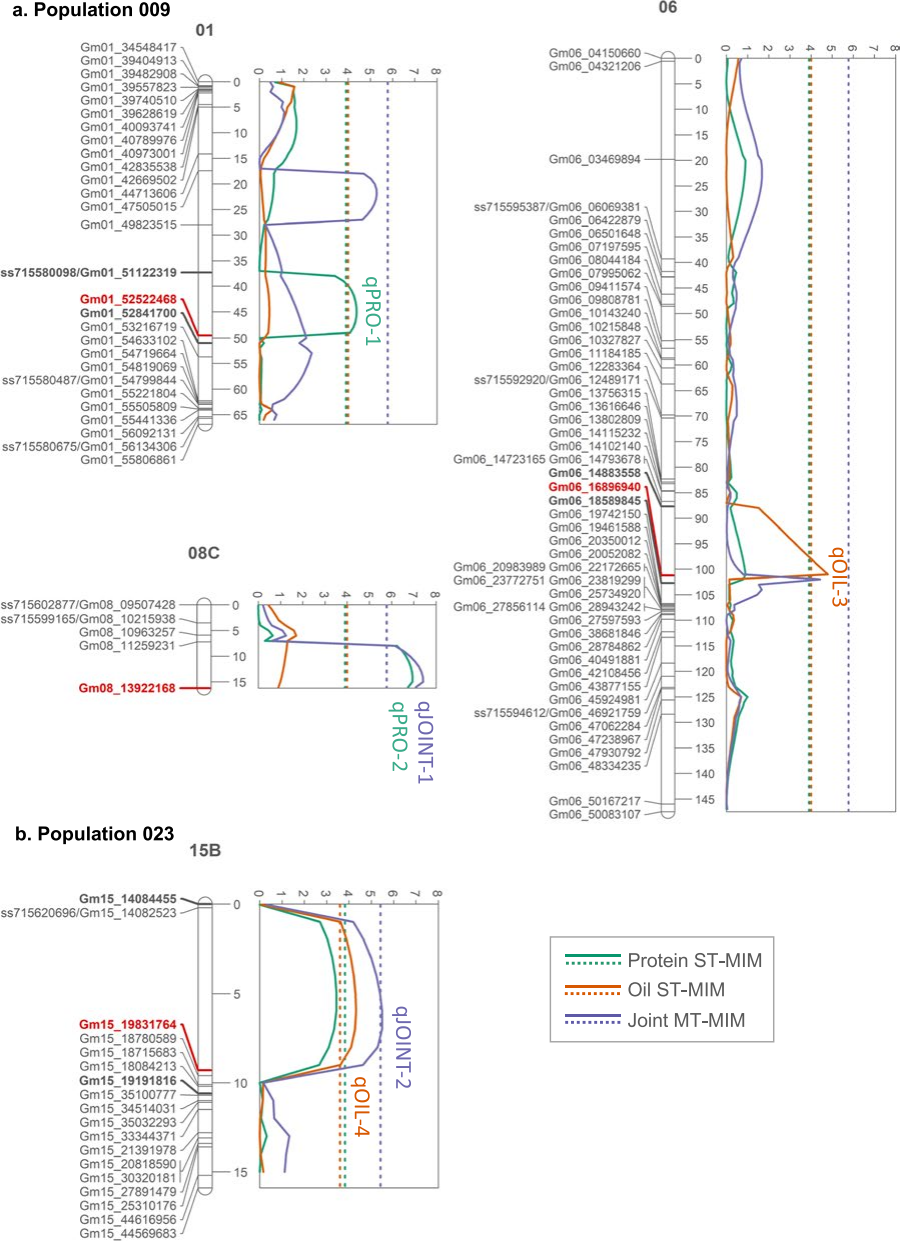

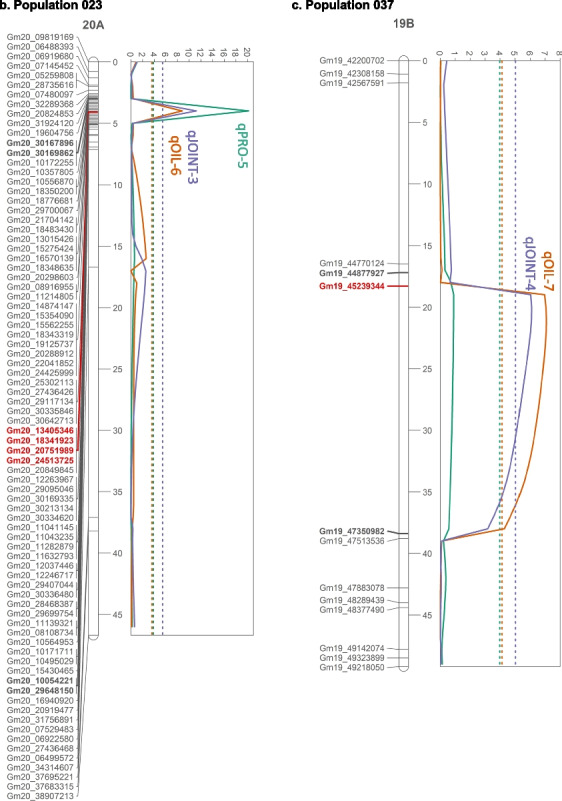
QTL results from single-trait MIM and multiple-trait MIM. The maps of linkage groups harboring significant QTLs were displayed for populations a) 009, b) 023, and c) 037. Solid lines indicate LOD score, and dotted lines LOD thresholds. Red bold texts indicate peak markers, and black bold texts indicate flanking markers associated with each QTL. Markers are named as GmX_Y where X is the chromosome number, and Y is the SNP’s physical position (in bp) in the Wm82 reference genome. SoySNP50K markers were included where present

Using ST-MIM, we identified seven QTLs across all populations (Table 3). For protein content, three QTLs were found on Chrs 1, 8, and 20; these consisted of qPRO-1 and qPRO-2 for population 009 and qPRO-4 for population 023. Four QTLs were detected for oil content. On LG 20A in population 023, qOIL-5 co-localized with qPRO-4. The QTLs qOIL-3 in population 009, qOIL-6 in population 023, and qOIL-7 in population 037 showed no significant associations with protein (Fig. 2a).

**Table 3:** Significant quantitative trait loci from single-trait MIM (ST-MIM) and multiple-trait MIM (MT-MIM) analyses

Population	Single-trait MIM									Corresponding multiple-trait MIM							
	Trait	QTL	LG^a^	LOD^b^	Peak pos^c^ (cM)	Peak marker^d^	CI^e^ (cM)	Flanking markers^d^	Genes^f^	QTL	LG^a^	LOD^b^	Peak pos^c^ (cM)	Peak marker^d^	CI^e^ (cM)	Flanking markers^d^	Genes^f^
009	Protein	qPRO-1	01	4.4 (4.0)	45	Gm01_52522468	37–50	Gm01_49823515, Gm01_53218170	386	–	–	–	–	–	–	–	–
		qPRO-2	08C	6.9 (4.0)	14	Gm08_13922168	7–16	Gm08_11259231, Gm08_13869567	263	qJOINT-1	08C	8.7 (5.8)	15	Gm08_13922168	7–16	Gm08_11259231, Gm08_13869567	263
	Oil	qOIL-3	06	4.8 (4.0)	101	Gm06_16896940	95–102	Gm06_14883558, Gm06_18590431	258	–	–	–	–	–	–	–	–
023	Protein	qPRO-4	20A	20.2 (3.8)	4	Gm20_18341923	3–5	Gm20_30167962, Gm20_10054221	310	qJOINT-2	20A	11.2 (5.4)	4	Gm20_18341923	3–5	Gm20_30167962, Gm20_10054221	310
	Oil	qOIL-5	20A	8.8 (3.6)	4	Gm20_18341923	3–5	Gm20_30167962, Gm20_10054221	310								
		qOIL-6	15B	4.3 (3.6)	6	Gm15_19831764	0–10	Gm15_14010941, Gm15_19191816	233	qJOINT-3	15B	5.5 (5.4)	6	Gm15_19831764	0–10	Gm15_14010941, Gm15_19191816	233
037	Oil	qOIL-7	19B	7.1 (4.1)	21	Gm19_45239344	18–35	Gm19_44877927, Gm19_47350982	302	qJOINT-4	19B	6.1 (5.7)	20	Gm19_45239344	18–34	Gm19_44877927, Gm19_47350982	302

Overlapping ST-MIM and MT-MIM loci are placed in the same row. Not all ST-MIM loci have a corresponding MT-MIM locus, and an absence of a MT-MIM locus is indicated as blank (–)

^a^LG: linkage group

^b^LOD cutoffs are shown in parentheses below the LOD score

^c^Peak pos: Peak position

^d^Markers are named in the format GmX_Y where X is the chromosome number and Y is the physical position (in bp) of the SNP on the Wm82 reference genome

^e^*CI *confidence interval

^f^Number of genes within the physical interval flanked by the flanking markers based on the Wms82.a2.v1 reference genome (www.soybase.org)

Using MT-MIM, we identified four QTLs, designated as qJOINT-1 to -4. The QTLs qJOINT-1, -3, and -4 co-localized with the single-trait QTLs qPRO-2 on LG 08C, qOIL-6 on LG 15B, and qOIL-7 on LG 19B, respectively, while the locus qJOINT-2 on LG 20A corresponded to two single-trait QTLs, qPRO-4 and qOIL-5. The single-trait loci of qPRO-1 and qOIL-3 did not co-localize with any multi-trait QTLs (Table 3). The multiple-trait approach of MT-MIM always had higher LOD thresholds than those of ST-MIM (Fig. 2, Table 3). Two ST-MIM loci, qPRO-1 and qOIL-3, were not detected by MT-MIM. Though analyses with MT-MIM revealed a peak co-localizing with the ST-MIM QTL in the case of qOIL-3 and a peak within 15 cM away from the ST-MIM QTL regarding qPRO-1, both of these MT-MIM peaks failed to reach the LOD thresholds and were not statistically significant (Fig. 2). Based on this observation, MT-MIM did not perform as well as ST-MIM in our study, leading to the rejection of our hypothesis on the superiority of MT-MIM over ST-MIM.

### Comparison of MT-MIM and ST-MIM performance using simulated populations with varied heritability levels and QTL allelic effects

Given that MT-MIM did not outperform ST-MIM in contrary to previous studies, we hypothesized that for traits with high heritability, MT-MIM may not offer advantages over ST-MIM. To test this proposition, we conducted a simulated study to assess how ST-MIM and MT-MIM performed in detecting QTLs linked to oil and protein traits at varied heritabilities.

Results obtained from the simulated data showed as the heritability levels increased, both ST-MIM and MT-MIM exhibited improved QTL detection capabilities. Furthermore, both ST-MIM and MT-MIM demonstrated superior performance in identifying major-effect QTLs compared to minor-effect ones. Both methods consistently and successfully detected major-effect loci, such as qSIM-3 and qSIM-5, with high probabilities ranging from 0.95 to 1.00 (Table 4).

**Table 4:** Phenotypic variance explained and additive effects of QTLs for seed oil and protein contents

Population	Locus (LG)	Phenotypic variance explained (%)		Additive effect^a^				Protein-to-oil change ratio
		Protein content	Oil content	Protein content (g/kg)	Oil content (g/kg)	EPV_NOPA_ bushel^−1^ ($)^b^	EPV_HY+Q_ bushel^−1^ ($)^b^	
009	qPRO-1 (LG 01)	11.2%	2.0%	+ 4.51	-0.94	-0.01	+ 0.04	-4.8
	qPRO-2 qJOINT-1 (LG 08C)	6.3%	2.8%	+ 5.21	-1.27	-0.02	+ 0.04	-4.1
	qOIL-3 (LG 06)	2.9%	11.5%	+ 1.83	-2.85	-0.04	-0.04	-0.6
023	qOIL-4 qJOINT-2 (LG 15B)	6.3%	6.5%	+ 3.13	-5.61	-0.08	-0.08	-0.6
	qPRO-5 qOIL-6 qJOINT-3 (LG 20A)	52.4%	54.6%	+ 5.91	-14.39	-0.21	-0.23	-0.4
037	qOIL-7 qJOINT-4 (LG 19B)	3.6%	20.4%	+ 2.12	-3.25	-0.05	-0.04	-0.7

^a^A positive-signed effect represents an increasing allele from the A parent; a negative-signed effect, an increasing allele from the B parent. Population 009: H09-730 (parent A) x N09-2516 (parent B); population 023: H09-018 (parent A) x N09-2495 (parent B); population 037: Summit (parent A) x N09-2516 (parent B)

^b^Additive effect on EPV was calculated as the difference in EPV values per bushel derived from the mean oil and protein contents between each allele

Total seed protein and oil contents were recorded in grams per kilogram of seed weight (g/kg)

However, at heritabilities 0.3, 0.5, and 0.7 with minor-effect loci, MT-MIM had an increased probability of detecting QTLs for each trait relative to ST-MIM. This is reflected in the case of qSIM-2, a locus designed to exert a minor effect on oil and a major effect on protein content. MT-MIM (*P* = 1.00) performed exceptionally better than ST-MIM of oil content, the trait with minor effects under scenarios HET0.3, HET0.5, and HET0.7 (*P* = 0.10, 0.25, 0.85, respectively). However, MT-MIM identified the qSIM-2 locus at the same rate as ST-MIM of protein, the trait with major effects (Table 4).

At a heritability of 0.9, MT-MIM offers no benefit, as it performs similarly to ST-MIM regardless of minor or major QTLs and pleiotropic effects. ST-MIM excelled in detecting even minor effect QTLs, such as qSIM-1, -2, and -4, with consistently high probabilities ranging from 0.95 to 1.00, performing similarly well compared to MT-MIM (Table 4).

### Allelic effects on oil and protein contents and the derived estimated processed values

The allelic effect and phenotypic variance explained by each QTL were extracted (Table 5). All QTLs showed opposite effects on oil and protein contents with varying protein-to-oil change ratios (Table 5). For population 009, the protein-associated QTL qPRO-1 and qPRO-2 had small effects on oil content (PVE = 2.0% and 2.8%) but a relatively large effect on protein content, explaining 11.2% and 6.3% of the phenotypic variance of protein content. For both loci, the allele from parent H09-730 increased protein by 4.5–5.2 g/kg while decreasing oil by 0.9–1.3 g/kg, displaying an approximate -4.8 and -4.1 ratio of protein to oil change, respectively. For population 023, the QTL on LG 20A explained an equally significant amount of phenotypic variance between 52.4% and 54.6% for protein and oil contents and showed a -0.4 ratio of protein to oil change. The QTL on LG 15B explained approximately 6.4% phenotypic variance for both traits. For population 037, the oil-associated QTL on LG 19B accounted for a major effect on oil content at 20.4% phenotypic variance but a relatively minor effect on protein content (3.6%). It displayed a -0.7 ratio of protein to oil change. Notably, the oil-increasing allele came from the high-yield parent Summit instead of the high-oil parent N09-2516 (Table 5).

**Table 5:** Probability of single- and multiple-trait multiple interval mapping correctly detecting simulated QTLs as significant at different heritability levels

Scenario	Heritability	Method	Probability of correctly detecting a QTL as significant				
			qSIM-1	qSIM-2	qSIM-3	qSIM-4	qSIM-5
HET0.3	0.3	Oil ST-MIM	0.05	0.10	1.00	0.00	–
		Protein ST-MIM	0.00	1.00	1.00	–	1.00
		Oil ST-MIM or Protein ST-MIM	0.05	1.00	1.00	0.00	1.00
		Joint MT-MIM	0.10	1.00	1.00	0.05	0.95
HET0.5	0.5	Oil ST-MIM	0.35	0.25	1.00	0.00	–
		Protein ST-MIM	0.05	1.00	1.00	–	1.00
		Oil ST-MIM or Protein ST-MIM	0.35	1.00	1.00	0.00	1.00
		Joint MT-MIM	0.35	1.00	1.00	0.00	1.00
HET0.7	0.7	Oil ST-MIM	0.80	0.85	1.00	0.75	–
		Protein ST-MIM	0.30	1.00	1.00	–	1.00
		Oil ST-MIM or Protein ST-MIM	0.80	1.00	1.00	0.75	1.00
		Joint MT-MIM	0.85	1.00	1.00	0.65	0.95
HET0.9	0.9	Oil ST-MIM	1.00	1.00	1.00	0.95	–
		Protein ST-MIM	0.95	1.00	1.00	–	1.00
		Oil ST-MIM or Protein ST-MIM	1.00	1.00	1.00	0.95	1.00
		Joint MT-MIM	1.00	1.00	1.00	0.95	1.00

Estimated processed values per bushel were calculated using two pricing methods, the capped method NOPA and the uncapped method HY + Q (Market View Insight No. 19–2: Overview of Soybean Meal Valuation Methods 2019). For the protein-associated QTLs on Chrs 01 and 08 in population 009, each oil-enhancing and protein-reducing allele from parent N09-2516 would increase the EPV_NOPA_ by $0.01–0.02 while decreasing the EPV_HY+Q_ by $0.04. For all the remaining QTLs, the oil-enhancing allele increased both EPV_HY+Q_ and EPV_NOPA_ to relatively the same extent. Because qJOINT-3 in population 023 had large effects on oil and protein contents (over 50% phenotypic variance explained) and the additive effect on oil content was substantial (± 1.4%), the oil-increasing allele increased EPV_NOPA_ and EPV_HY+Q_ by a noticeable amount of $0.21 and $0.23, respectively (Table 5).

### Candidate genes functioning in seed oil and protein production and accumulation

A candidate gene search was conducted for each QTL, and genes involved in protein and oil biosynthesis and accumulation were identified for each QTL. The results showed that qJOINT-1 on Chr 01 had 10; qPRO-2 and qJOINT-1 on Chr 08 had 18; qOIL-3 on Chr 06 had 9; qPRO-4, qOIL-5, and qJOINT-2 on Chr 20 had 12; qOIL-6 and qJOINT-3 on Chr 15 had 7; and qOIL-7 and qJOINT-4 on Chr 19 had 20 candidate genes (Table 6).

**Table 6:** Candidate genes for each QTL based on predicted function and physical position

QTL	Gene ID	Functional annotation	Biological process
qPRO-1	***Glyma.01G159800***^***a***^	Phosphoserine aminotransferase	L-serine biosynthetic process
	***Glyma.01G160000***	Serine acetyltransferase	cysteine biosynthetic process from serine
	*Glyma.01G161100*	Lysine histidine transporter-like protein	amino acid transport
	*Glyma.01G162100*	Phospholipase D	phosphatidic acid metabolic process
	*Glyma.01G162800*	Aconitate hydratase	glycolysis
	*Glyma.01G163000*	Aspartic proteinase-like protein	amino acid transport
	*Glyma.01G164000*	Cornifelin	cysteine biosynthetic process
	*Glyma.01G164300*	3-dehydroquinate synthase	aromatic amino acid family biosynthetic process
	*Glyma.01G194700*	N-acetyl-l-glutamate synthase	arginine biosynthetic process
	*Glyma.01G197700*	Malate dehydrogenase	fatty acid beta-oxidation
qPRO-2, qJOINT-1	*Glyma.08G148000*	Peptide transporter	lipid transport; peptide transport
	*Glyma.08G148500*	Basic helix-loop-helix transcription factor	fatty acid beta-oxidation
	*Glyma.08G150800*	Uroporphyrinogen-III C-methyltransferase	threonine catabolic process
	*Glyma.08G151800*	Anthranilate phosphoribosyltransferase-like protein	tryptophan biosynthetic process
	***Glyma.08G152200***	Lipid-transfer protein/seed storage 2S albumin superfamily protein	lipid transport
	*Glyma.08G157700*	Citrate synthase	glycolysis
	*Glyma.08G159900*	ATP-citrate synthase	cysteine biosynthetic process; fatty acid biosynthetic process
	*Glyma.08G160500*	Isocitrate dehydrogenase	gluconeogenesis; glycolysis
	*Glyma.08G161600*	Carboxyl-terminal peptidase	cysteine biosynthetic process
	***Glyma.08G161700***	Lipid-transfer protein/seed storage 2S albumin superfamily protein	lipid transport
	*Glyma.08G164100*	Inosine-5'-monophosphate dehydrogenase	methionine biosynthetic process
	***Glyma.08G164600***	Cysteine synthase	cysteine biosynthetic process from serine; serine family amino acid metabolic process; sulfur amino acid metabolic process
	*Glyma.08G165400*	Phosphoglycerate kinase	glycolysis
	*Glyma.08G165500*	Phosphoglycerate kinase	glycolysis
	*Glyma.08G167700*	Lipase	fatty acid beta-oxidation
	*Glyma.08G169400*	NAC domain protein	proline biosynthetic process
	*Glyma.08G172200*	Lojap-like protein	aromatic amino acid family biosynthetic process
	*Glyma.08G173700*	Photosystem II subunit R	cysteine biosynthetic process
qOIL-3	*Glyma.06G179200*	Galactinol-sucrose galactosyltransferase	sucrose biosynthetic process
	*Glyma.06G181600*	Phosphate/phosphoenolpyruvate translocator	phosphoenolpyruvate:phosphate antiporter activity
	*Glyma.06G184400*	Leucine-rich repeat transmembrane protein kinase	acetyl-CoA metabolic process; starch metabolic process
	*Glyma.06G184700*	GDSL-motif lipase	lipid metabolic process
	*Glyma.06G185500*	Protein farnesyltransferase subunit	fatty acid beta-oxidation
	*Glyma.06G185700*	GDSL-motif lipase	lipid metabolic process
	***Glyma.06G194500***	GDSL-motif lipase	lipid metabolic process
	***Glyma.06G200200***	Sugar transporter	fructose transport
	***Glyma.06G200800***	Sugar transporter GmSWEET19	fructose transport
qPRO-4, qOIL-5, qJOINT-2	*Glyma.20G049800*	Bifunctional inhibitor/lipid-transfer protein/seed storage 2S albumin superfamily protein	lipid transport; very long-chain fatty acid metabolic process
	*Glyma.20G053200*	Homoserine dehydrogenase	aspartate family amino acid biosynthetic process
	*Glyma.20G053700*	Lipoxygenase	aromatic amino acid family metabolic process; unsaturated fatty acid biosynthetic process
	*Glyma.20G054000*	Lipoxygenase	aromatic amino acid family metabolic process; unsaturated fatty acid biosynthetic process
	*Glyma.20G054100*	Lipoxygenase	aromatic amino acid family metabolic process; unsaturated fatty acid biosynthetic process
	*Glyma.20G055900*	Methionine synthase	methionine biosynthetic process
	*Glyma.20G058700*	GDSL esterase/lipase	lipid metabolic process
	*Glyma.20G060100*	Long-chain-fatty-acid-CoA ligase	long-chain fatty acid metabolic process
	*Glyma.20G060300*	Long-chain-fatty-acid-CoA ligase	long-chain fatty acid metabolic process
	*Glyma.20G062700*	Bifunctional inhibitor/lipid-transfer protein/seed storage 2S albumin superfamily protein	lipid transport
	*Glyma.20G064000*	GDSL esterase/lipase	lipid metabolic process
	*Glyma.20G064200*	Amino acid permease	amino acid transmembrane transport
qOIL-6, qJOINT-3	*Glyma.15G176000*	Transcription factor MYB121	fatty acid biosynthetic process
	***Glyma.15G181500***	Beta-ketoacyl-ACP synthase II	fatty acid biosynthetic process
	*Glyma.15G182900*	GDSL-motif lipase	amino acid transport; lipid metabolic process
	*Glyma.15G183000*	GDSL-motif lipase	amino acid transport; lipid metabolic process
	*Glyma.15G184300*	Pectinesterase	amino acid transport
	*Glyma.15G184400*	Pectinesterase	amino acid transport
	*Glyma.15G186400*	Aspartic proteinase	glycolysis; lipid metabolic process
qOIL-7, qJOINT-4	*Glyma.19G193500*	GDSL-motif lipase	lipid metabolic process
	*Glyma.19G194500*	Basic-leucine zipper (bZIP) transcription factor family protein	lipid storage
	*Glyma.19G197900*	Alpha/beta-hydrolases superfamily protein	methionine biosynthetic process
	*Glyma.19G198500*	Aspartyl protease family protein	glycine catabolic process
	*Glyma.19G199300*	BEL1-like homeodomain transcription factor	cysteine biosynthetic process
	*Glyma.19G199500*	BEL1-like homeodomain transcription factor	cysteine biosynthetic process
	*Glyma.19G200000*	Digalactosyldiacylglycerol synthase	unsaturated fatty acid biosynthetic process; lipid transport
	*Glyma.19G202900*	Alpha/beta-hydrolases superfamily protein	amino acid transport
	*Glyma.19G203200*	Aromatic and neutral transporter	amino acid transport
	*Glyma.19G209400*	Dephospho-CoA kinase	proline transport
	*Glyma.19G209700*	Amino acid dehydrogenase family protein	cysteine biosynthetic process
	***Glyma.19G212800***	Sucrose synthase	sucrose biosynthetic process
	*Glyma.19G215200*	Alpha/beta-hydrolases superfamily protein	lipid metabolic process
	*Glyma.19G218300*	Long-chain-fatty-acid-CoA ligase	fatty acid biosynthetic process; fatty acid metabolic process
	*Glyma.19G219300*	PII/GLNB1 protein	glycine catabolic process; regulation of fatty acid biosynthetic process
	*Glyma.19G221800*	Class II chitinase	amino acid transport
	*Glyma.19G224300*	Germin-like protein	amino acid transport
	*Glyma.19G226900*	GDSL-motif lipase	glycerol biosynthetic process; lipid metabolic process
	*Glyma.19G228200*	Galactose oxidase/kelch repeat superfamily protein	lipid transport
	*Glyma.19G229000*	Protein disulfide isomerase S-2	glycolysis; gluconeogenesis

^a^Genes in bold had literature evidence supporting their potential roles in controlling oil and protein contents (see discussion section)

## Discussions

### Detected QTLs confirmed previously identified oil and/or protein QTLs

QTL positions in this study were positioned on the consensus genetic map relative to the loci from previous seed oil and protein QTL analyses and meta-QTL analyses (Qi et al. 2018; Van and McHale 2017). All QTLs identified in this study overlapped with previously identified meta-QTLs and QTLs. In population 009, qPRO-1 on LG 01 overlapped with MQTLPro-1 and MQTLOil-2, while qPRO-2 and qJOINT-1 on LG 08C overlapped with MQTLOil-28 (Qi et al. 2018). In population 037, qOIL-7 on LG 19B overlapped with MQTLPro-48, MQTLOil-59 (Qi et al. 2018), and mPO19-5 (Van and McHale 2017).

The QTL regions projected to Chr 15 from population 023 did not overlap with any meta-QTLs. However, a search of QTL projected to the same physical regions on SoyBase revealed that qOIL-4 and qJOINT-2 on LG 15B overlapped with previously mapped protein-associated QTLs Seed protein 41–2 (Jun et al. 2008) and 4–13 (Lee et al. 1996). Regarding LG 20A in the same population, the interval of qPRO-4, qOIL-5, and qJOINT-3 flanked by markers at physical positions of 10,054,221 and 30,167,962 bp spans a large genomic region. When projected to the same physical map on SoyBase, this region overlapped with meta-QTLs mPO20-3, -4, and mO20-3 (Van and McHale 2017). It also overlapped with previously mapped oil and protein QTLs, including Seed oil 14–3 (Csanádi et al. 2001), 39–10 (Wang et al. 2014), 42–16, 42–18, 42–37, 42–38 (Han et al. 2015) and Seed protein 34–11 (Lu et al. 2013), 37–7, 37–9 (Wang et al. 2014), 41–4 (Jun et al. 2008), and 26–4 (Reinprecht et al. 2006). Despite these coincident QTLs, it is important to note that markers on both LGs 15B and 20A from the population 023 genetic map are not in the same linear order as the Wm82 reference genome sequence, explaining huge physical intervals derived from the flanking markers in both cases (Table 3). Because the LGs 15B and 20A genetic markers have some discrepancies relative to expectations (Fig. 2b) and the confidence interval of each QTL is projected to the reference genome using the relative position of the marker on the reference genome Wm82, the true position of the QTL relative to the Wm82 reference genome and other studies may not be accurate. Overall, although this study did not detect novel QTLs, genetic regions previously associated with seed oil and protein contents were confirmed, underscoring QTL stability across genetic backgrounds and environments.

### Multiple-trait QTL analysis did not offer improvement from the single-trait QTL approach for our traits

Several methods for multiple-trait QTL analysis have been developed, including maximum likelihood multivariate regression, least-squares multivariate regression, dimension reduction techniques like principal component analysis or discriminant analysis, Markov chain Monte Carlo algorithm, and Bayesian statistics (Alam et al. 2021; Banerjee et al. 2008; Mi et al. 2010; Singh and Singh 2015; Xu et al. 2009). However, there is a limited number of publicly accessible packages and software, such as R/qtlbim, R/rmtqtl, QGene, and Windows QTL Cartographer. These tools often support only a restricted set of common mapping population types, such as RIL, double haploid, backcross, and F2 populations.

In our study, we opted for the maximum likelihood approach, MT-MIM, due to its comparability with the equivalent single-trait approach, ST-MIM, its availability to the public, and its ability to handle RIL populations. A maximum likelihood approach, the MT-MIM method is built upon the mechanism of ST-MIM using the models of multiple-trait composite interval mapping (MT-CIM), an extension of composite interval mapping (CIM) for multiple traits. MT-MIM possesses the accuracy of MIM and the sensitivity of the multiple-trait approach without being too computationally expensive (Joehanes 2009). Multiple-trait approaches like MT-MIM have been claimed to improve the precision and power of single-trait analyses by producing high LOD scores, correcting and consolidating disparate single-trait peaks into a single precise joint-trait peak, and producing sharper peaks or smaller intervals (Costa E Silva et al. 2012; Joehanes 2009).

While previous studies showed that MT-MIM had improved power to detect QTL in comparison to single-trait methods, from our empirical studies, MT-MIM confirmed only five of the seven single-trait QTLs and did not offer much improvement in power and mapping accuracy compared to ST-MIM. We hypothesized that our findings diverged from previous studies because, for high heritability traits like seed oil and protein contents (*H*^*2*^ = 0.84–0.94 in our study), the advantage of using the multiple-trait approach over the single-trait one diminishes. To test our hypothesis, we utilized simulations to evaluate the effectiveness of ST-MIM and MT-MIM in handling traits with various heritability levels, specifically 0.3, 0.5, 0.7, and 0.9. We simulated three multiple-trait QTLs: qSIM-1, which had minor effects on both traits; qSIM-2, with a minor effect on one trait and a major effect on the other; and qSIM-3, which had major effects on both traits. Additionally, we introduced two single-trait QTLs, qSIM-4 and qSIM-5, each with minor and major effects, respectively, on only one trait (Table 1). These combinations allowed us to assess whether MT-MIM could outperform ST-MIM by identifying any minor effect QTLs that ST-MIM might miss. For the lower heritability scenarios HET0.3, HET0.5, and HET0.7, MT-MIM offers higher detective ability over ST-MIM in identifying minor effect QTLs. For the highest heritability scenario of HET0.9, which most resembles our empirical traits, ST-MIM and MT-MIM performed equally well, consistently detecting all QTLs with probabilities between 0.95 and 1.00 (Table 4). Our findings suggested that for high heritability traits like seed oil and protein contents, single-trait analyses are sufficient for identifying minor- and major-effect QTLs.

### The QTLs exhibited varied protein-to-oil change ratios, offering opportunities to strategically optimize these traits

Although the negative correlation between oil and protein content in soybeans poses challenges for simultaneously enhancing both traits, breeding programs can leverage variations in this correlation to produce soybean seeds with optimized economic and nutritional values. Typically observed across various commercial cultivars and diverse germplasm, a 1 percentage point increase in oil content leads to a decrease in protein content by just under 2 to 3 percentage points (Kambhampati et al. 2019; Lee et al. 2019). This relationship is quantified by a protein-to-oil change ratio of approximately -2 to -3. Despite this consistent pattern, variations in these ratios offer opportunities to enhance the processed value of soybeans.

In our study, all identified QTLs demonstrated an inverse effect on oil and protein contents, reflecting the well-established relationship between these two traits. However, the magnitude of their effects varied significantly, as evidenced by the protein-to-oil additive effect ratios, which ranged from -0.4 to -4.8 (Table 4). Notably, these ratios deviated from the typical -2 to -3 range. For example, QTLs qPRO-1 and qPRO-2 exhibited high protein-to-oil change ratios of -4.1 and -4.8, indicating that a substantial increase in protein content by 4 to 5 percentage points would minimally impact the oil content, decreasing it by just 1 percentage point. Conversely, QTLs like qOIL-3, qPRO-4, qOIL-5, qOIL-6, and qOIL-7, with ratios between -0.4 to -0.7, suggest that a 1 percentage point increase in oil would only result in a 0.5 percentage point decrease in protein. Overall, the diverse effects of different QTLs on oil and protein contents offer breeders valuable targets for selective breeding, enabling strategic manipulation to achieve specific breeding objectives and enhance the overall processed value of soybeans.

### Allelic effects on estimated processed values varied based on the calculation methods

Estimated processed values per bushel can be calculated in this study using two methods: NOPA and HY + Q. Both methods consider yield, oil, and protein contents and multiply them by market prices to calculate EPV. The two methods of calculating EPV differ only in determining the price of meal protein content. Meal protein values are calculated from and higher than whole seed protein contents. The capped NOPA method imposes a penalty in price for protein concentrations below 48% and does not offer a premium for concentrations above 48%. Therefore, the relationship between meal protein content and price is only positively linear until the meal protein value reaches 48%, then price plateaus despite further increases in meal protein content. In contrast, for the uncapped HY + Q method, prices continue to increase consistently and linearly as meal protein increases past 48%. Thus, HY + Q offers more rewards as protein content elevates and prices the same meal protein value higher than NOPA (Market View Insight No. 19-2: Overview of Soybean Meal Valuation Methods 2019).

The different meal value calculation approach between the NOPA and HY + Q methods, along with the scale of the protein-to-oil change ratios, explain why for the protein QTLs on Chrs 1 and 8 from population 009, the oil-enhancing allele would increase the EPV_NOPA_ and decrease the EPV_HY+Q_, while for the other five QTLs, the oil-enhancing allele increased both EPV_HY+Q_ and EPV_NOPA_. Both alleles of all QTLs rendered meal protein content above the 48% ceiling, and thus, meal protein price was capped at $353.02/ton for EPV_NOPA_ (data not shown), leading to oil content driving changes in EPV_NOPA_ for the studied populations and explaining why the oil-enhancing or protein-reducing allele would always increase EPV_NOPA_ per bushel. The protein QTLs on Chrs 1 and 8 both had high protein-to-oil change ratios of -4.1 and -4.8 (Table 3), meaning an increase in oil leads to a four- to five-time decrease in protein. This small increment in oil content leads to a slight increase in oil value, which cannot offset the large reduction in meal value caused by the accompanying substantial linear reduction in protein contents and leads to a decrease in EPV_HY+Q_ per bushel. For all other QTLs from populations 023 and 037 as well as qOIL-3 from population 009, since the protein-to-oil change is relatively small and below 1 (-0.4 to -0.7, Table 3), meaning a two-time elevation in oil content is accompanied with a one-time reduction in protein content as well as meal content. Hence, the oil-enhancing allele would lead to a larger increase in oil value that could offset the smaller diminution in meal value and thus improve the final EPV_HY+Q_ per bushel. The oil-increasing alleles of the QTLs on Chrs 15, 19, and 20 are the alleles harbored in the reference genome Wm82.

### Candidate genes involved in seed oil and protein production and accumulation were identified

We conducted a candidate gene search within the genomic regions of each QTL (Table 5). Some candidate genes have strong evidence for their functions in seed oil and protein production and accumulation. Within the Chr 01 region where qPRO-1 was projected to, *Glyma.01G160000*, predicted to encode a *serine acetyltransferase,* is linked to sulfur assimilation, which plays a role in the accumulation of the seed storage protein β subunits. *Glyma.01G160000* was 5.3-fold upregulated in lines with low seed β subunit content (Zhang et al. 2019). *Glyma.01G159800*, predicted to encode a phosphoserine aminotransferase enzyme involved in serine biosynthesis, was identified as a candidate gene in a photosynthesis-associated genome-wide association study (Zhong et al. 2018). Its Arabidopsis and wheat homologs are engaged in photosynthesis and nitrogen assimilation, and their elevated expression in turn altered the amino acid profile and total storage protein content in wheat grain and Arabidopsis and induced starch accumulation in transgenic duckweed (*Lemna turionifera)* (Wang et al. 2022; Wulfert and Krueger 2018; Zhong et al. 2018).

For qOIL-3 projected to Chr 06, *Glyma.06G194500*, a GDSL-motif lipase/hydrolase family gene, is linked to the regulation of seed lipid metabolism and oil content in plants (Chen et al. 2012; Ding et al. 2019; Huang et al. 2015; Wei et al. 2008). Its homolog in Arabidopsis, *AT1G74460,* is expressed highly during embryo development (www.arabidopsis.org). The *bidirectional sugar transporter* genes *Glyma.06G200200* and *Glyma.06G200800* are 81% identical in sequence. *Glyma.06G200800* or *GmSWEET19* was categorized in the same clades of the Arabidopsis *SWEET16* and *SWEET17* (Hooker et al. 2022), which facilitate bidirectional fructose transport across the vacuolar membrane in root and leaf cells (Guo et al. 2014).

The locus of qPRO-2 and qJOINT-1 on Chr 8 contains *Glyma.08G152200* and *Glyma.08G161700*, both annotated as seed storage proteins in the seed storage 2S albumin superfamily*. Seed storage 2S albumin* was identified as a hub gene for soybean seed storage composition during seed filling using chromosome segment substitution lines. *Seed storage 2S albumin* was upregulated in the high protein high oil line and the low protein high oil line while downregulated in the high protein low oil line (Qi et al. 2018). Additionally, Glyma.08G164600, a *cysteine synthase*, is involved in sulfur assimilation and cysteine biosynthesis and plays a role in regulating seed storage protein synthesis in cereal grains and soybean (Anderson and Fitzgerald 2001; Kim et al. 1999). *Glyma.08G164600* is a potential target of the oil production-regulating transcription factor *WRINKLED1* (Arias et al. 2022).

Located within the interval of qOIL-6 and qJOINT-3 on Chr 15, *Glyma.15G181500* was annotated as an β-ketoacyl-acyl-carrier synthase II (*KASII)* enzyme, potentially functioning in the elongation of palmitic acid into stearic acid during fatty acid biosynthesis. Several mutations on two copies of KASII, *Glyma.17g047000* and *Glyma.13g112700*, have been identified to associate with significantly increased palmitic acid level and in rare cases, a decreased total oil content (Devereaux et al. 2024).

Overall, the candidate gene suggestions are based on literature research and provide potential targets for further characterization of the QTLs. Additional assessments, such as fine mapping, gene expression profiling, or generating transgenic lines targeting each gene of interest, are needed to confirm these genes as the underlying genetic controls of the QTLs.

In conclusion, our study identified seven QTLs linked to seed oil and protein contents, along with their potential candidate genes. These QTLs overlap with previous meta-QTLs or QTLs associated with seed oil and protein contents or compositions, indicating stability across environments and genetic backgrounds. Comparing the performance of single- and multiple-trait QTL analyses showed that, contrary to previous studies, MT-MIM did not significantly enhance the detection of QTLs for high heritability traits, like seed oil and protein contents, compared to ST-MIM. This study is the first to evaluate the use of the multiple-trait QTL analysis on the traits of soybean oil and protein contents, concluding that single-trait analyses were sufficient in identifying both minor and major effect QTLs. Furthermore, the assessment of allelic effects on EPV using two standard EPV calculation methods offers practical insights into the economic values of the QTLs. The use of RIL populations derived from advanced breeding line backgrounds enables the facile development of heterogeneous inbred families to determine the effect of each allele on yield and subsequently select and integrate those conferring desired outcomes into breeding programs.

### Supplementary Information

Below is the link to the electronic supplementary material.

Supplementary file1 (XLSX 196 KB)

Supplementary file2 (TXT 33 KB)

## Data Availability

All data in this project are made publicly available. The genotyping-by-sequencing (GBS) or genotypic data is available on NCBI Sequence Read Archive under the BioProject ID of PRJNA1048467. The phenotypic data (Table S1), the genetic map data (Tables S2-S4), and the scripts for processing and analyzing the GBS data are included in the Supplemental Information.
